# Harnessing bioactive nanomaterials in modulating tumor glycolysis-associated metabolism

**DOI:** 10.1186/s12951-022-01740-y

**Published:** 2022-12-12

**Authors:** Zhengying Gu, Chengzhong Yu

**Affiliations:** 1 School of Chemistry and Molecular Engineering, East China Normal University, Shanghai, 200241 People’s Republic of China; 2 Australian Institute for Bioengineering and Nanotechnology, The University of Queensland, Brisbane, QLD 4072 Australia

**Keywords:** Bioactive nanomaterials, Tumor metabolism, Glycolysis, Tumor immunity, Cancer therapy

## Abstract

Glycolytic reprogramming is emerging as a hallmark of various cancers and a promising therapeutic target. Nanotechnology is revolutionizing the anti-tumor therapeutic approaches associated with glycolysis. Finely controlled chemical composition and nanostructure provide nanomaterials unique advantages, enabling an excellent platform for integrated drug delivery, biochemical modulation and combination therapy. Recent studies have shown promising potential of nanotherapeutic strategies in modulating tumor glycolytic metabolism alone or in combination with other treatments such as chemotherapy, radiotherapy and immunotherapy. To foster more innovation in this cutting-edge and interdisciplinary field, this review summarizes recent understandings of the origin and development of tumor glycolysis, then provides the latest advances in how nanomaterials modulate tumor glycolysis-related metabolism. The interplay of nanochemistry, metabolism and immunity is highlighted. Ultimately, the challenges and opportunities are presented.

## Introduction

Reprogramming of glucose metabolism plays a key role in tumorigenesis [1, 2]. Cancer cells are more “glucose-starved” than normal cells due to the famous “Warburg effect” [3]. This effect leads most cancer cells to use aerobic glycolysis rather than oxidative phosphorylation (OXPHOS) to ensure the high energy and metabolite demands in cancer cells [4, 5]. The glycolysis is a series of sequential enzymatic reactions that convert glucose into the high-energy adenosine triphosphate (ATP) with lactate as a preferred product [4]. Extensive studies have shown that glycolysis signalling and its by-products significantly influence the interaction between cancer cells and host cells, playing a key role in driving tumor progression and metastasis. They are also crucial in shaping tumor immunosuppression [6, 7]. Over the past decade, there has been an ongoing interest in tumor glycolytic metabolism and a growing consensus that glycolytic dependence is a promising therapeutic target across diverse cancer cells [8–14]. To date, several metabolic inhibitors or regulators designed to target the core set of glycolytic signaling have advanced into clinical trials [15, 16]. However, the complexity of the tumor environment still limits the use of these promising new agents.

To address this challenge, bioactive nanomaterials are rapidly developing into new cancer therapeutic strategies via glycolytic regulation. Nanomedicine has already been applied in regulating other types of metabolisms, e.g., lipid metabolism, iron metabolism, autophagy and glutaminolysis [17–20]. The concept of harnessing nanotechnology in modulating biological targets and biochemical reactions is also advancing into nanomedicines that target tumor glycolysis. Recently, various nanomaterials such as metal-based inorganic/organic nanoparticles and polymer composites have been designed to modulate the upstream and/or downstream of glycolytic signaling, and to reverse immunosuppressive microenvironment in tumor. Studies have also demonstrated the feasibility of combining glycolysis modulation with other therapeutic approaches. Nevertheless, a dedicated review on the latest achievements in this cutting-edge and interdisciplinary field is still lacking to our knowledge.

Herein, we provide a timely summary of the latest progresses and understandings of glycolytic metabolism in tumor development and immunity. Then, an in-depth review is provided on the current strategies, utilities and limitations of bioactive nanomedicines that modulate tumor glycolytic pathways and metabolic phenotypes. Lastly, our perspectives on the challenges and future opportunities are also provided to advance the development of anti-tumor metabolic nanomedicines.

## Causes of tumor glycolysis

### Hypoxia and HIF-1

**Fig. 1:**
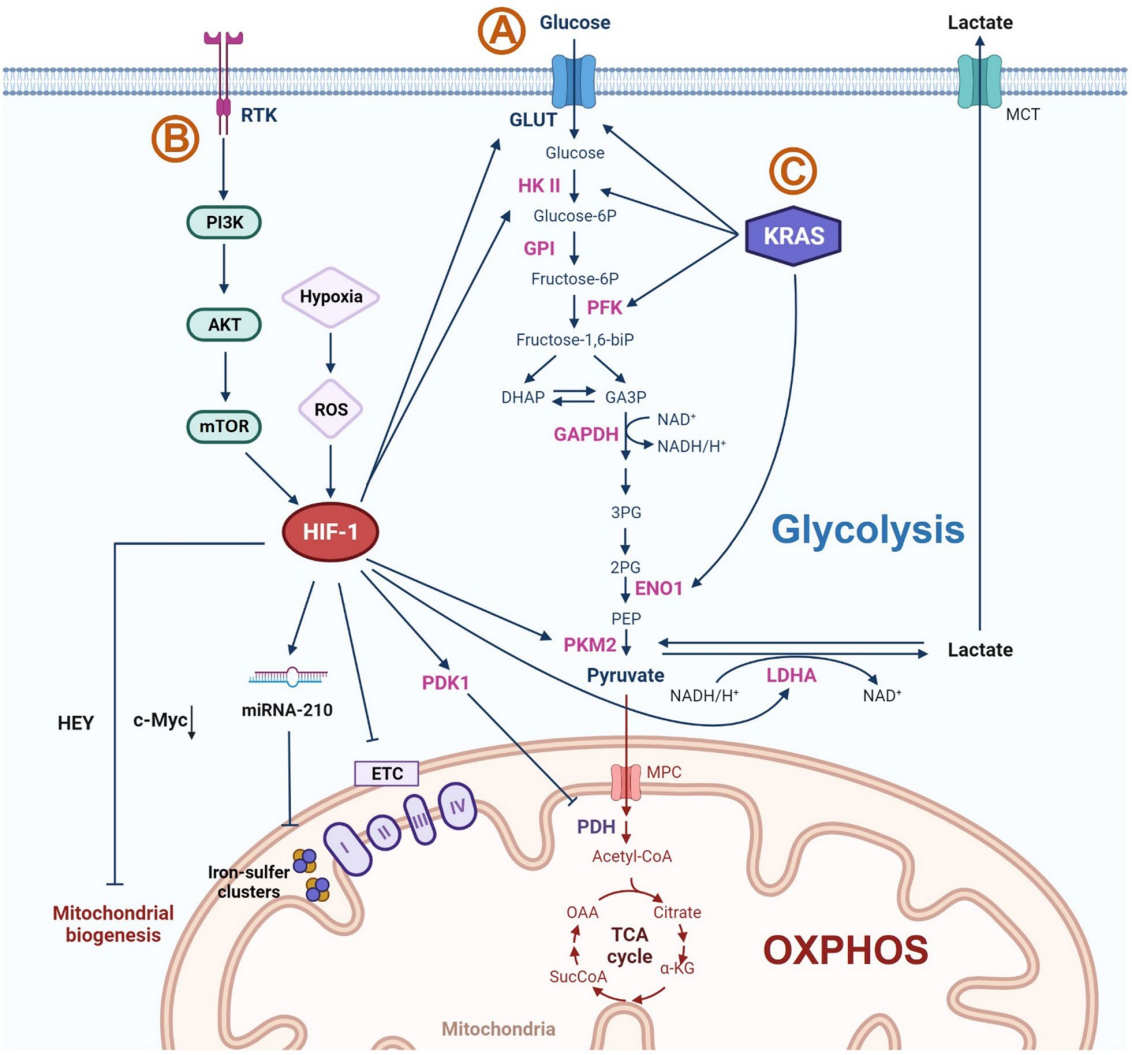
Key signalling pathways supporting glycolysis in tumor cells. **A** Hypoxia and HIF1α signalling dominates glycolysis in tumor cells via regulating multiple signalling pathways involved in both glycolytic and OXPHOS metabolisms. HIF-1 promotes glycolysis through increasing glucose influx and upregulating the expression of glycolytic enzymes. Meanwhile, it suppresses OXPHOS via disrupting mitochondrial functions and biogenesis. **B** The famous carcinogenic RTK-PI3K–AKT–mTORC1 signalling pathway interplays with HIF-1 to promote glycolytic metabolism. **C** Oncogenic mutation such as KRAS stimulates glucose uptake and facilitates glycolytic activities. *Glucose-6P* glucose-6-phosphate; *Fructose-6P* fructose-6-phosphate; *Fructose-1,6-biP* fructose 1,6- bis phosphatase; *DHAP* dihydroxyacetone phosphate; *GA3P* glyceraldehyde 3-phosphate; *NAD+*  nicotinamide adenine dinucleotide; *NADH* nicotinamide adenine dinucleotide (NAD)+ hydrogen (H); *3PG* 3-phosphoglycerate; *2PG* 2-phosphoglycerate; *PEP* phosphoenolpyruvate; *MPC* mitochondrial pyruvate carrier; *Acetyl-CoA* Acetyl-coenzyme A; *OAA* oxaloacetate; *SucCoA* succinyl-coenzyme A; *α-KG* alpha-ketoglutarate; *ETC* electron transport chain; *c-Myc* cellular myelocytomatosis ongogene; *MCT* monocarboxylate transporters. Image was created with www.BioRender.com

The glycolytic process begins with the uptake of glucose and culminates in the production of pyruvate through successive enzymatic reactions (Fig. 1A) [4, 19]. Pyruvate is either reduced to lactate via lactate dehydrogenase A (LDHA), or enters into the TCA cycle within mitochondria via pyruvate dehydrogenase (PDH) for OXPHOS under aerobic conditions. At this fork, the hypoxia-inducible factor 1 (HIF-1) directs cancer cells towards the glycolytic pathways by mastering the transcriptional regulation of adaptive response to hypoxia [21, 22].

HIF-1 is an oxygen-dependent transcriptional regulator that is sensitive to reactive oxygen species (ROS) [23]. It is stimulated by ROS accumulation and energy consumption resulting from hypoxia and rapid tumor growth [24]. HIF-1 elevates glucose uptake through upregulating the expression of glucose transporter (GLUT) [1, 25], and potentiates the transcription of glycolytic enzymes such as hexokinase II (HK II) [26–28], pyruvate dehydrogenase kinase (PDK) [29], pyruvate kinase M2 (PKM2) [30, 31] and lactate dehydrogenase (LDH) [21, 31]. These promoted enzymes in turn maintain the HIF-1activity [12].

HIF-1 also facilitates glycolysis via impairing mitochondrial function, such as deactivation of tricarboxylic acid cycle (TCA cycle) and inhibition of mitochondrial biogenesis [21, 32]. Earlier studies have shown that HIF-1 enhanced activation of PDK1 suppresses the activity of pyruvate dehydrogenase (PDH), which is the key enzyme for pyruvate oxidation and initiation of TCA cycle [33]. Meanwhile, HIF-1 decreases mitochondrial activity by disrupting electron transfer. This not only deactivates subunits of electron transport chain (ETC) (e.g., complex 1 and complex 4) [34, 35], but also induces micro-RNA-210 (miR-210) to repress the assembly of iron-sulfur clusters that functions as electron transfer groups [36–38]. In addition, HIF-1 disrupts mitochondrial biogenesis. For example, it inhibits the expression of c-Myc proteins that increases the number of mitochondria in cells [39]. Recently, it has been demonstrated that the generation of healthy mitochondria is further repressed under hypoxia via Hes-related family BHLH transcription factor with YRPW motif (HEY) [34].

### Other regulators

In addition to the master regulator HIF-1, an increasing number of regulators have been identified to drive tumor glycolysis. These regulators vary from kinases to oncogenes, which have been well reviewed [12, 14, 40]. Here, we only show two representative regulators. The first one is RTK-PI3K–AKT–mTORC1 signaling pathway (Fig. 1B). It is one of the most attractive therapeutic target candidates that has a significant role in carcinogenesis, which involves the promotion of glycolysis [41, 42]. Receptor tyrosine kinases (RTKs) are transmembrane proteins overexpressed in a variety of cancers [43]. They stimulate phosphoinositide 3-kinases (PI3K) which recruit and activate RAC (Rho family)-alpha serine/threonine-protein kinases (AKT) [44–46]. These kinases are responsible for cell growth, survival and proliferation, being able to alter metabolisms in cancer cells. AKT activation promotes glucose influx by upregulation of the expression of glucose transporters and activation of glycolytic enzymes [47, 48]. More importantly, AKT leads to the activation of downstream mammalian target of rapamycin (mTOR), which acts as a central activator of the Warburg effect under normoxic conditions [49]. Active mTOR upregulates HIF-1 through Forhead box protein K1 (Foxk1) [50]. It also mediates regulatory effects on the expression of a serious glycolytic enzymes and proteins including GLUT, HK II, PFK and PKM2 glycolysis via HIF and MYC-dependent signaling, further interacting with the aforementioned glycolytic pathways [50].

Kirsten rat sarcoma viral oncogene homologue (KRAS), a famous highly mutated oncogene in all cancers, has been recognized as another important glycolytic regulator (Fig. 1C) [51, 52]. Mutant KRAS deregulates glycolysis in multiple ways. It promotes glycolytic activities via positively regulating relevant transporters and proteins (e.g., GLUT1, HK, LDH, Phosphofructokinase (PFK), and alpha-enolase-1 (ENO1)) [52] as well as negatively altering mitochondrial functions [53]. Thus, cancer cells with high level of RAS mutation are highly vulnerable to glycolytic inhibitors, as evidenced by the downregulation of glycolytic enzyme glyceraldehyde 3-phosphate dehydrogenase (GAPDH) and impaired tumor growth in vitamin C treated KRAS mutant mouse intestinal cancers [52].

In addition to above signaling pathways, the biosynthetic requirements of tumor during rapid growth also contribute to glycolysis [54, 55]. Although the ATP yield in aerobic glycolysis is lower than that in OXPHOS, the faster rate of ATP production by glycolysis can meet the demands of rapid tumor cell division [55]. Moreover, the glycolytic intermediates provide cancer cells precursors (e.g., nucleotides, amino acids and lipids) to build essential macromolecules (e.g., DNA, RNA, proteins, and lipids) for proliferation [55]. On top of that, the by-products such as lactate in turn support tumor progression and stable the glycolytic flux [56]. These metabolic demands beyond ATP production promote tumor to rely on glycolysis.

To date, the cause of tumor glycolysis has not yet been fully understood and remains an important research direction. Nevertheless, the increased efforts to understand the causes and underlying mechanisms of tumor glycolysis provide new therapeutic opportunities to target weakness and susceptibility in glycolysis-driven cancers cells.

## Effects of glycolysis on tumor development and immunity

### Growth

**Fig. 2:**
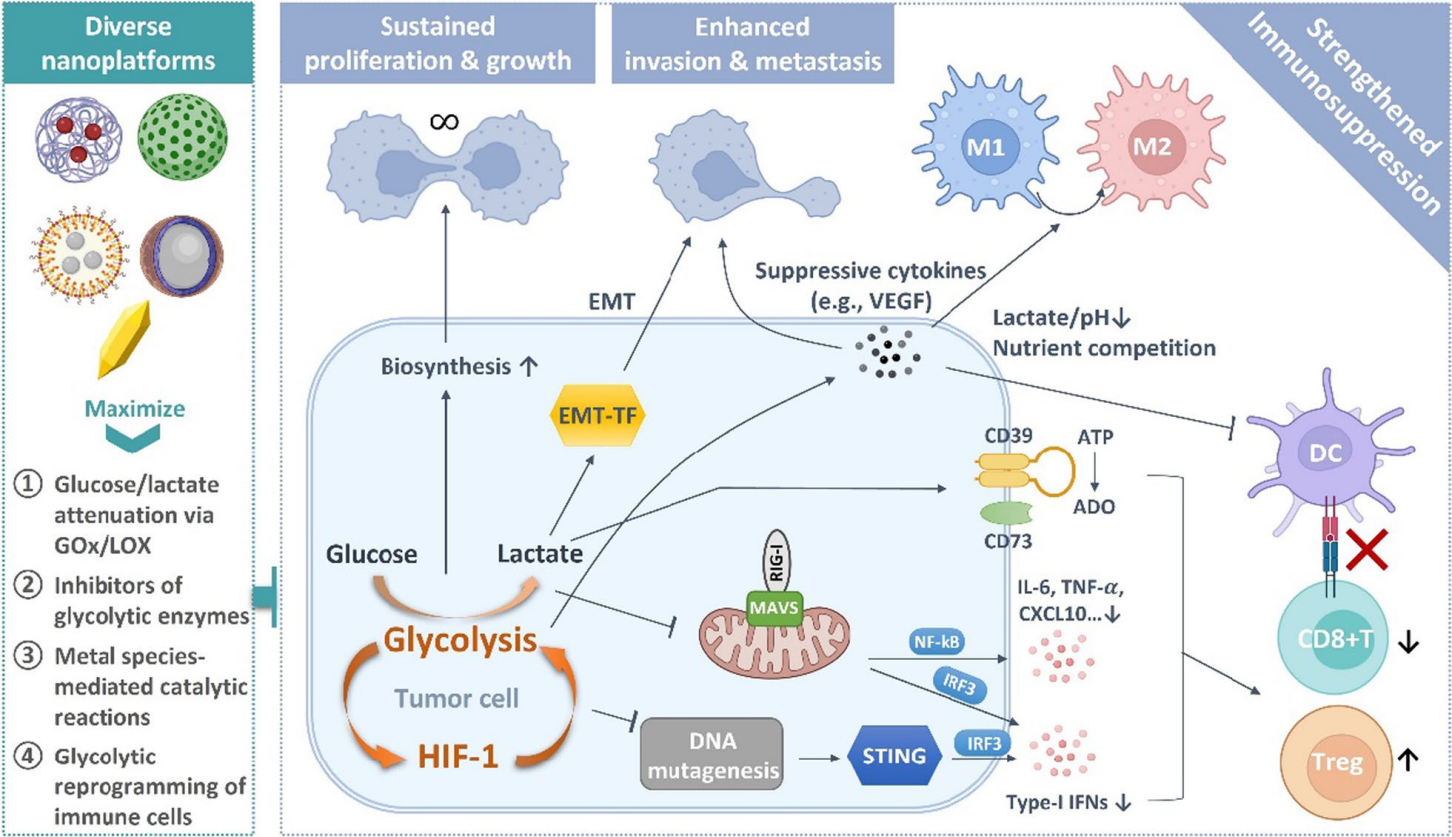
Mutually enhanced glycolysis and hypoxia in tumor synergize to promote tumor proliferation, invasion and metastasis, and to suppress anti-tumor immunity through multiple pathways. Diverse nanotherapeutic platforms are developed to regulate tumor glycolysis generally via four strategies

The high rate of glycolysis favors tumorigenesis and malignancy progression (Fig. 2). It allows cancer cells to expand the use of nutrients and energy which are required for rapid biosynthetic activities. [8, 12] The yield of ATP per glucose consumed in glycolysis is nearly 18 time less efficient compared to OXPHOS, however, the rate of ATP generation is much more rapid (approximately 100 times faster) [57]. High glycolytic flux offers cancer cells sufficient metabolic intermediates to meet biosynthetic demands and rapid proliferation, such as ribose sugars, glycerol, citrate, amino acids, and nicotinamide adenine dinucleotide phosphate (NADPH) [58].

### Metastasis

Enhanced glycolysis in hypoxia promotes tumor invasion and metastasis. The uncontrolled proliferation of cancer cells in hypoxia environment results in extreme nutrient-deficiency [59, 60]. To satisfy the energy demand, the activities of glycolytic enzymes and production of lactate are extensively enhanced. These glycolytic intermediates regulate tumor metastasis via epithelial–mesenchymal transition (EMT), angiogenesis and colonization [61]. EMT is a key mechanism that regulates all stages of cancer progression from initiation, invasion, metastasis to colonization [62]. EMT endows cancer cells with mesenchymal cell characteristics in which both migratory capacity and resistance to apoptosis are greatly enhanced [63, 64]. Glycolysis promotes EMT process by facilitating EMT-inducing transcription factor (EMT-TFs) expression with the assistance of lactate and reduced pH in tumor microenvironment [61, 65]. Glycolysis is also critical in angiogenesis, in which it upregulates production of vascular endothelial growth factor (VEGF) to promote vessel sprouting [61, 66, 67]. Moreover, glycolysis is also important in preserving stemness and inducing differentiation of cancer stem cells [68–70]. The secretion of lactate protects cancer stem cells from toxicity of nature killer (NK) cells, further promoting metastatic colonization [61].

### Immunosuppression

Glycolysis has been shown to suppress tumor immunogenicity and aid tumor immune escape. It has been reported that the glycolytic by-product lactate impedes retinoic acid-inducible gene I (RIG-I) like receptors (RLRs) mediated type-I IFNs production, impairing cancer immunosurveillance [71]. There are two members of RLRs that can sense cytosolic RNA to trigger innate immune response [72, 73]. They are RIG-I and melanoma differentiation-associated protein 5 (MDA5). Once activated, these two receptors interact with the protein of mitochondrial antiviral-signaling (MAVS) protein, followed by activation of Interferon regulatory factor 3 (IRF3) and nuclear factor kappa-light-chain-enhancer of activated B cells (NF-$\kappa$ B) and subsequent transcription of type-I IFNs and other inflammatory cytokines and chemokines such as interleukin 6 (IL-6) and tumor necrosis factor alpha (TNF-$\alpha$) [71, 73, 74]. These inflammatory factors recruit and activate dendritic cells (DCs) and effector CD8 + T cells in tumor site [75, 76]. Lactate is able to bind to MAVS and disrupt MAVS-RIG-I interaction, thereby impairing anti-tumor immunity. The type-I IFN secretion may also be reduced through downregulation of the stimulator of interferon genes (STING) signaling resulting from aerobic glycolysis [77]. STING signaling serves as a central role in anti-tumor immune signaling cascade, which can be stimulated by aberrant cytosolic double-strand DNA (dsDNA) [78]. Vardhana et al. proposed that the shift from OXPHOS to glycolysis may result in reduced DNA mutagenesis which thereby depress STING signaling [77].

In addition, glycolytic tumor further inhibits antitumor immunity while promote immunosuppressive phenotype of tumor associated immune cells through hypoxia and lactic acidosis [6]. For instance, the tumor immunity is suppressed via the activities of HIF-1-dependent CD39 and CD73 in glycolytic tumor. HIF-1 upregulates the expression of cell-surface ectonucleotidases CD39 and CD73 [79, 80], which convert immunostimulatory ATP into adenosine (ADO), an immunosuppressive metabolite that remarkably dampens immune responses varying from impeding DC maturation and effector cell function to stabilizing suppressive regulatory cells [81, 82]. It has been proved that T cell effector functions impaired by tumor glycolysis results in immune resistance to adoptive T cell therapy. [83] Immunostimulatory signals such as interferon regulatory factor 1 (IRF1) and C-X-C motif chemokine ligand 10 (CXCL10) are reduced in glycolytic tumors that suppress cytotoxicity of T cell. The lactic acidosis has been found to impair the frequency and function of plasmacytoid DCs that are associated with patient overall survival in metastatic melanoma [84]. As both the functions of DCs and effector T cells are inhibited in glycolytic tumors, it is suggested that tumor specific antigen presentation, a key step of anti-tumor immunity occurring between DC and T cells, is dramatically impeded. Lactate also facilitate establishment of immunosuppressive microenvironment via promoting generation of suppressive M2 macrophages and regulatory T cells (Tregs) [85, 86]. It is worthy to note that glycolysis is also essential in the activation of anti-tumor CD8 + cytotoxic T cells and pro-inflammatory macrophages [87–89]. As a result, glycolytic tumor cells with a high rate of proliferation may compete with these immune cells for key nutrients, further restricting stimulation of anti-tumor immunity.

## Nanotherapeutic strategies

As glycolytic activities significantly impact on tumor progression, the development of nanotherapeutic strategies to interrupt tumor glycolysis has gained ongoing efforts (Fig. 2). Glucose is the source of glycolytic activities while the downstream lactate drives tumor development in multiple ways. These two molecules therefore become preferred targets for nanotherapeutic exploration.

### Direct regulation aiming at glucose starvation and lactate attenuation

Metal–organic frameworks (MOFs) have attracted increasing attention as a versatile platform in biomedical application [90, 91]. These porous coordination polymers are constructed from metal ions and functional organic ligands with the unique properties of easy functionalization, high porosity, cargo loading capacity and tunable biocompatibility. Zinc-based zeolitic imidazolate frameworks (ZIFs) are a representative paradigm of MOFs with ZIF-8 as a typical example [92], which has been used as a promising nanotherapeutic agent for glycolysis-associated anti-tumor therapies. Due to the mild synthetic conditions, ZIF-8 can encapsulate drug molecules or biomolecules such as proteins and nucleic acids in situ during the synthesis [93]. It also shows pH-responsive property, enabling on- demand release of cargo molecules and zinc ions (Zn^2+^) in acidic tumoral and intracellular environment [94–96].

**Fig. 3:**
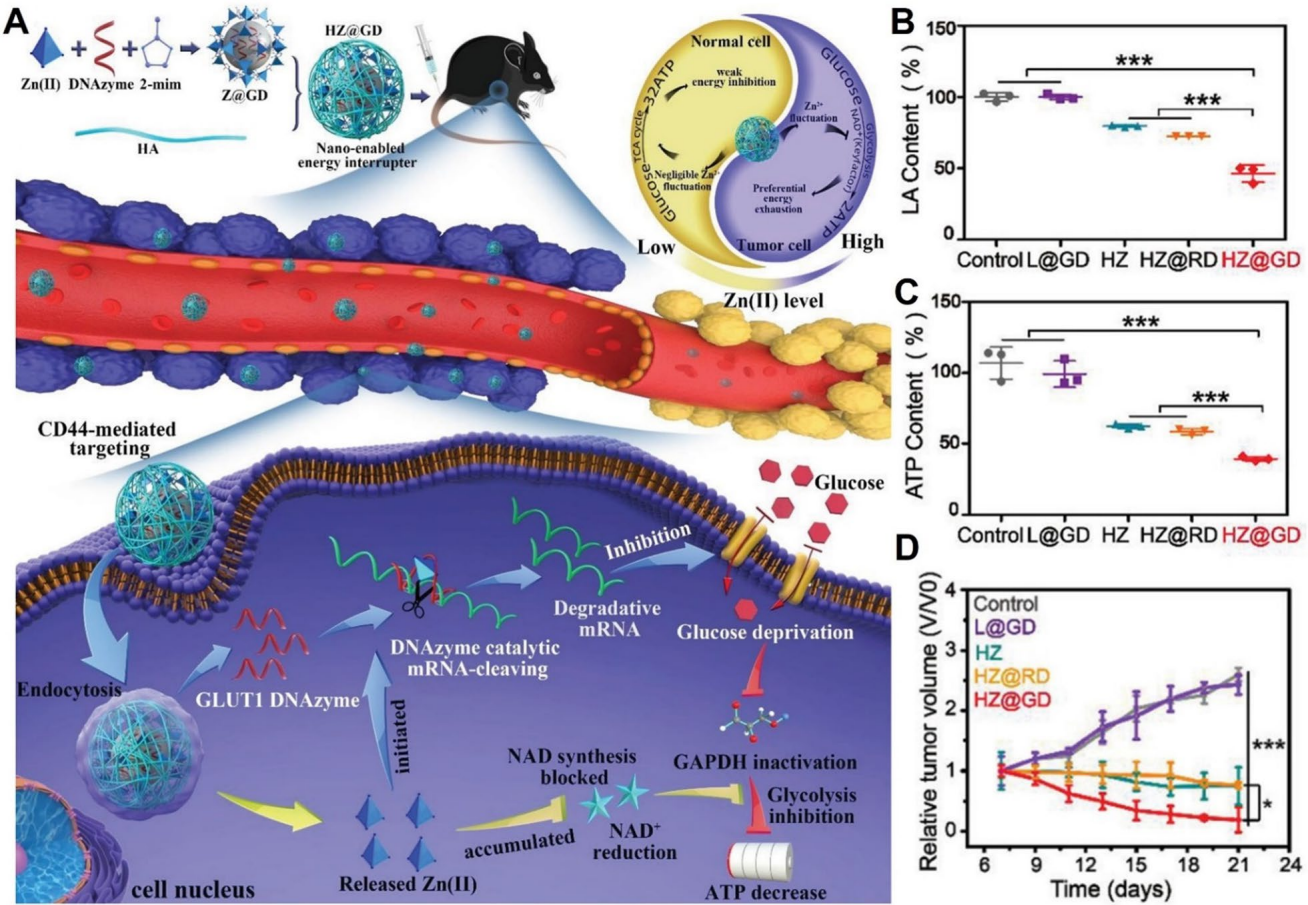
**A** Schematic presentation of two-pronged glucose starvation therapy based on DNAzymes-loaded ZIF-8 nanocomposites. **B** Intratumoral lactate and **C** ATP levels after treatment. **D** Tumor growth profiles. Reproduced with permission [97]. Copyright © 2021, Wiley‐VCH GmbH

Taken advantages of these merits, Wu et al. utilized hyaluronic acid (HA)-coated ZIF-8 to deliver deoxyribozymes (DNAzymes) that targets GLUT1 to tumor site (Fig. 3A). [97] This nanoparticle preferentially accumulated in tumor via a CD44-mediated active targeting mechanism. Both Zn^2+^ and DNAzymes were released in response to intracellular hyaluronidase and acidic environment. Interestingly, intracellular Zn^2+^ overload was found to inhibit glycolysis pathway through blocking the synthesis of NAD + and subsequently inactivating GAPDH, demonstrating the promising potential of ZIF-8 in serving as nanotherapeutic agents for tumor glycolytic regulation. Meanwhile, DNAzymes was stimulated by the high level of intracellular Zn^2+^, cleaving GLUT1 mRNA to cut off glucose supply. Through these synergistic effects, intratumoral lactate and ATP levels decreased (Fig. 3B, C). The nanocomposites achieved potent starvation effects on melanoma in a mutually reinforcing manner (Fig. 3D) with relatively low toxicity on melanocytes, providing a promising glucose starvation strategy.

**Fig. 4:**
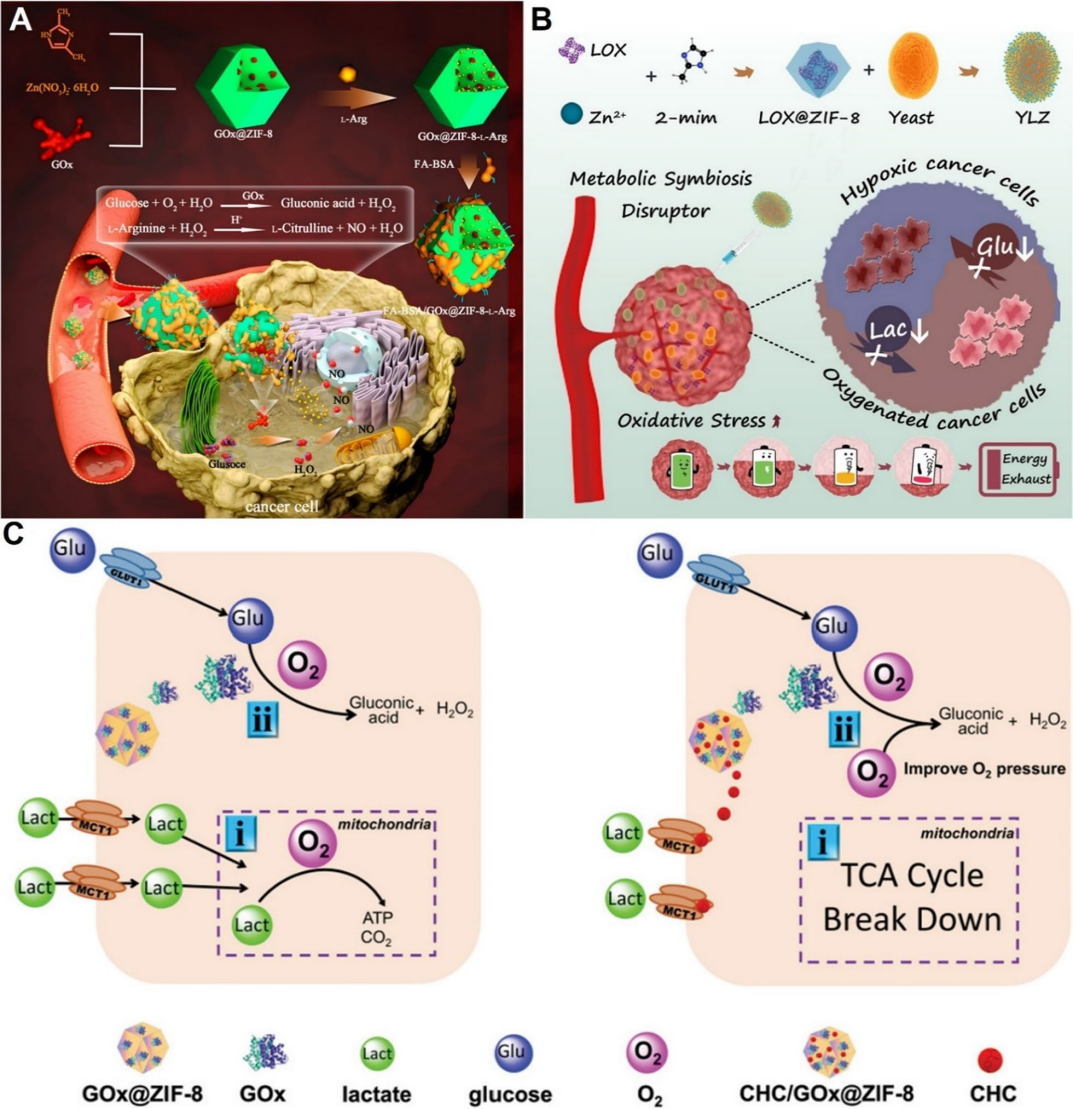
**A** Scheme of the ZIF-8 based glucose starvation therapy in combination with NO therapy [98]. Copyright © 2022 American Chemical Society. **B** Illustration of the synergistic glucose and lactate attenuation via Yeast@LOX@ZIF-8 nanoparticles [103]. Copyright © 2021 Elsevier Ltd. **C** Mechanism of how blockade of lactate influx in tumor cells promotes glucose starvation therapy [105]. Copyright © 2021 Wiley–VCH GmbH

ZIF-8 nanoparticles can also efficiently deliver glucose oxidase (GOx) and lactate oxidase (LOX) to tumor for glucose and lactate depletion [98–105]. Catalyzed by GOx, glucose can be converted into gluconic acid and hydrogen peroxide (H_2_O_2_) [98]. Liu et al. decorated GOx-loaded ZIF-8 nanoparticles (GOx@ZIF-8) with L-Arginine, which could react with H_2_O_2_ to form nitric oxide (NO), dramatically enhancing the anti-tumor effect of glucose starvation therapy (Fig. 4A) [98]. In addition to GOx-induced catalytic reaction, glucose can also be depleted by non-pathogenic yeasts in hypoxia conditions [103]. Wang et al. grafted LOX-loaded ZIF-8 nanoparticles (LOX@ZIF-8) to the surface of live yeasts through amide formation reaction to achieve synergistic glucose and lactate attenuation (Fig. 4B) [103]. Notably, the viability of yeasts after decoration of nanoparticles can be maintained for several days. Although the duration is sufficient to complete validation in animal models, it may limit future bulk storage and clinical applications.

Yu et al. demonstrated that blocking lactate influx was another effective strategy to enhance the therapeutic effects of glucose starvation therapy (Fig. 4C) [105]. α-cyano-4-hydroxycinnamate (CHC) is an inhibitor of monocarboxylate transporters (MCTs) that are responsible for transporting of lactate. Due to the strong affinity between zinc ions and carboxyl groups, the CHC and GOx molecules were encapsulated in ZIF-8 nanoparticles during synthetic process. It was found that CHC-mediated blocking of lactate influx could break down TCA cycle and reduce consumption of O_2_ in mitochondria. The elevation of oxygen not only relieved hypoxia but also promoted conversion from glucose to gluconic acid, significantly enhanced anti-tumor activities.

It has been demonstrated that increased oxidative stress facilitated ZIF-8-induced starvation therapy to inhibit tumor progression [101, 104]. Although excessive zinc ions can cause oxidative stress, the level may be limited as zinc ions are redox inert in biology [106]. Intracellular levels of ROS can be boosted by embedding redox-active metal oxides such as manganese dioxide (MnO_2_) and magnetite (Fe_3_O_4_) in the structure of ZIF-8 nanoparticles [101, 104]. MnO_2_ produced free radicals through redox reaction with the high level of intracellular glutathione (GSH) and H_2_O_2_, while Fe_3_O_4_ generated ROS by releasing ferrous and ferric ions to perform well-known Fenton reactions.

**Fig. 5:**
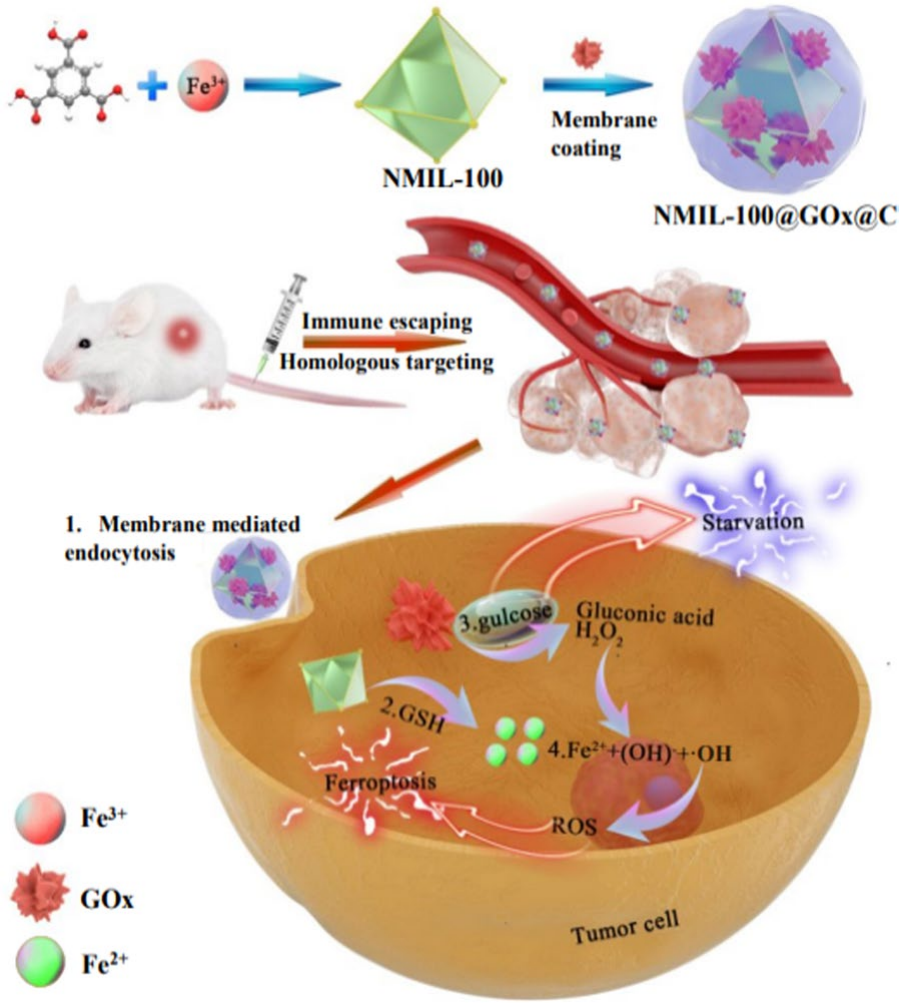
Schematic illustration of cancer cell membrane-coated iron-based MOF nanoparticles for synergistic ferroptosis and glucose starvation therapy. Cited with permission [107]. Copyright © 2020 American Chemical Society

Unlike ZIF-8, iron-based MOF nanoparticles can induce excessive oxidative stress by themselves to synergize with starvation therapy. Wan et al. utilized cancer cell membrane-coated iron-based MOF (namely NMIL-100) nanoparticles to deliver GOx (Fig. 5) [107]. The cancer cell membrane coating directed nanoparticles to target tumor sites with high efficiency. After internalized by cancer cells, NMIL-100 nanoparticles collapsed in response to acidic intracellular environment and high level of GSH to release iron species and GOx by the follow Eq. 1. Then, the GOx catalysed the depletion of glucose and production of H_2_O_2_ (Eq. 2). The latter involved in Fenton reaction of Fe^2+^ to generate highly toxic ^⦁^OH radicals which induced ferroptosis in tumor (Eq. 3).

1 $${\mathrm{Fe}}^{3+}+\mathrm{GSH}\to {\mathrm{Fe}}^{2+}+\mathrm{GSSG},$$

2 $$\mathrm{glucose}+ {O}_{2}\stackrel{\mathrm{GO}x}{\to }\mathrm{gluconic\,acid}+ {\mathrm{H}}_{2}{\mathrm{O}}_{2},$$

3 $${\text{Fe}}^{2 + } + {\text{H}}_{2} {\text{O}}_{2} \to {\text{Fe}}^{3 + } + \left( {{\text{OH}}} \right)^{ - } + {}^{ \cdot }{\text{OH }}$$

By exploiting synergetic ferroptosis and glucose starvation, the therapeutic nanoparticles demonstrated excellent tumor suppression performance. In addition to glucose oxidation reactions, the iron-dependent radical production can also be improved by enhancement of intracellular acidity [108]. Prussian blue nanoparticles (PBNM), another iron-based MOF [109, 110], have exhibited pH-selective catalytic activities. They can generate ^·^OH radicals under acidic pH while produce O_2_ under normal or alkaline pH conditions. To strengthen ROS generation, Wang et al. induced intracellular lactate accumulation and enhanced acidity by blocking the MCTs on cancer cells [108]. This method was less toxic to normal cells because MCT expression of normal cells was relatively lower than that of cancer cells.

**Fig. 6:**
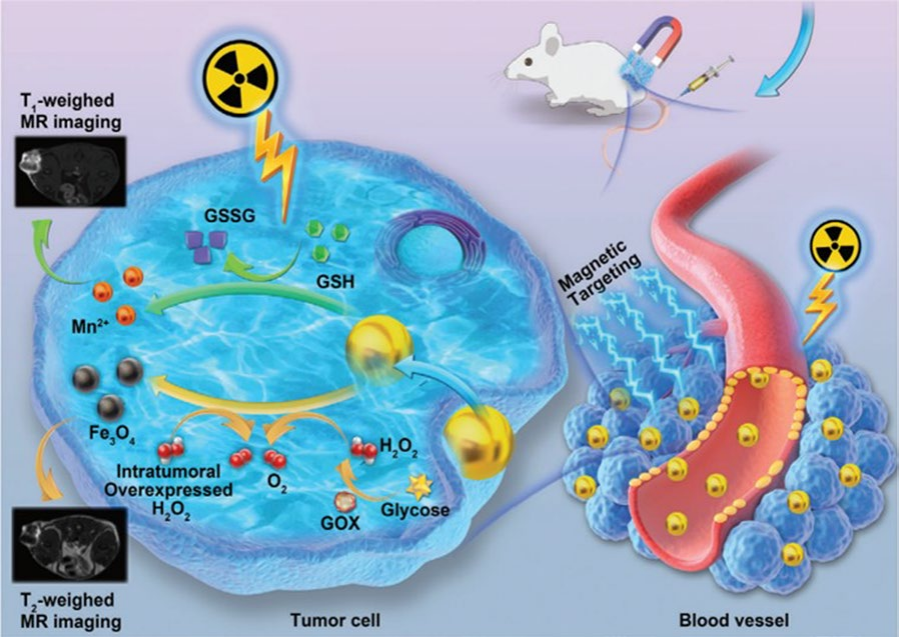
Illustrative scheme of Fe_3_O_4_@MnO_2_ nanoparticles for radiation enhancement and glucose starvation therapy. Cited with permission [113]. Copyright © 2020 WILEY–VCH Verlag GmbH & Co. KGaA, Weinheim

Similar regulating effects of GSH-dependent glucose depletion and ROS-induced cytotoxicity on tumor cells can be obtained by other metal-based inorganic nanoparticles in combination with GOx [111–114]. For example, Cu^2+^ ions released from a copper-embedded hollow mesoporous silica (HMSN-Cu) nanoparticles [112] and Mn^4+^ ions produced from Fe_3_O_4_@MnO_2_ nanoparticles [113] (Fig. 6) can oxidize the GSH into glutathione disulfide (GSSG), favouring the catalytic activities of GOx. Notably, the MnO_2_ shell induced GSH oxidation in the latter case was found to suppress the self-repair of DNA double-strand which often occurs in cancerous cells after radiotherapy. Furthermore, the Fe_3_O_4_@MnO_2_ was able to generate oxygen to relieve hypoxia via decomposition of H_2_O_2_ produced by reaction between GOx and glucose. In addition, Mn^2+^ ions reduced from MnO_2_ and Fe_3_O_4_ can be utilized for T_1_-weighed and T_2_-weighed contrast agents, respectively. Thus, the Fe–Mn bimetallic nanoparticles were able to enhance radiotherapy and magnetic resonance imaging simultaneously in addition to regulation of glucose metabolism. [113]

**Fig. 7:**
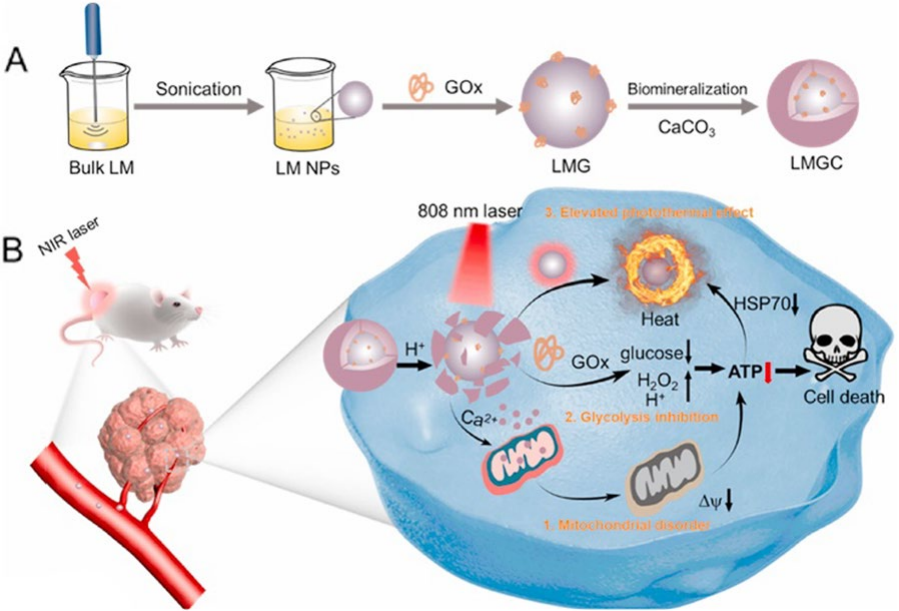
Schematic diagram of combination therapy of PTT and starvation therapy via liquid metal-CaCO_3_ nanocomposites. LM, liquid metal; LMG, LM nanoparticles coated with GOx; LMGC, LMG mineralized by amorphous calcium carbonate. Reproduced with permission. Copyright © 2022 Elsevier Ltd

Beside Fenton catalytic metal species, GOx can also synergize with oxidatively inert metal ions, such as Ca^2+^, to inhibit glycolysis in tumor. Ding et al. synthesized liquid metal nanoparticles decorated with GOx and calcium carbonate (CaCO_3_) to cut off ATP supply via GOx-inhibited glycolysis and Ca^2+^-induced mitochondrial dysfunction (Fig. 7) [114]. Disrupting Ca^2+^ homeostasis by using calcium-based nanoparticles (e.g., CaP and CaCO_3_) has been proved to be an effective strategy to damage mitochondria and trigger cancer cell apoptosis [115, 116]. In the design of Ding’s nanoparticle, gallium-indium liquid metal was used to perform photothermal therapy (PTT) [114]. Usually after PTT treatment, the damaged tumor cells produced heat shock proteins (HSPs) in large quantities to protect themselves from hyperthermia [117, 118]. It was found that Ca^2+^/GOx-induced reduction of energy supply markedly suppressed the expression of HSPs, demonstrating the potential of combined metabolic modulation and PTT [114]. The results are consistent with another report in which HSP inhibitor and GOx together enhanced the efficacy of mild-temperature PTT [119]. It has been demonstrated that Ca^.2+^ overload-induced mitochondrial disruption promotes autophagy [120]. Wang et al. reported the blockade of autophagosome degradation via obatoclax could inhibit ATP release, synergizing with GOx to cut off energy sources in starvation therapy [120].

In the absence of synergistic effects of metal species, the therapeutic effects of GOx can be achieved by improved GOx delivery and protection via rational design of delivery systems. Zeng et al. prepared dissolving microneedles containing GOx-loaded polydopamine (PDA) nanoparticles for localized melanoma therapy [121]. This delivery system achieved a high capsulation efficiency (58%) and long-lasting catalytic activities of GOx in local environment. The PDA nanovehicles facilitated GOx to retain activity up to 6 d under different incubation conditions (e.g. 10% serum, cell lysate and PBS buffer). Due to the self-dissolving property, the microneedles achieved highly efficient topical delivery of GOx to melanoma tumor without causing adverse effects. This GOx-delivery system showed 91% of inhibition rate in a mouse melanoma tumor model.

Nanomaterials-induced blockade of tumor blood capillaries is another useful strategy to achieve starvation therapy. Shi et al. designed a magnesium silicide (Mg_2_Si) nanoparticle which releases silane and reacts with oxygen in acidic tumor microenvironment to form silicate aggregates [122]. This in situ formation of silicate aggregates effectively cut off the supply of oxygen and nutrients to tumor, which may limit tumor glycolysis.

**Fig. 8:**
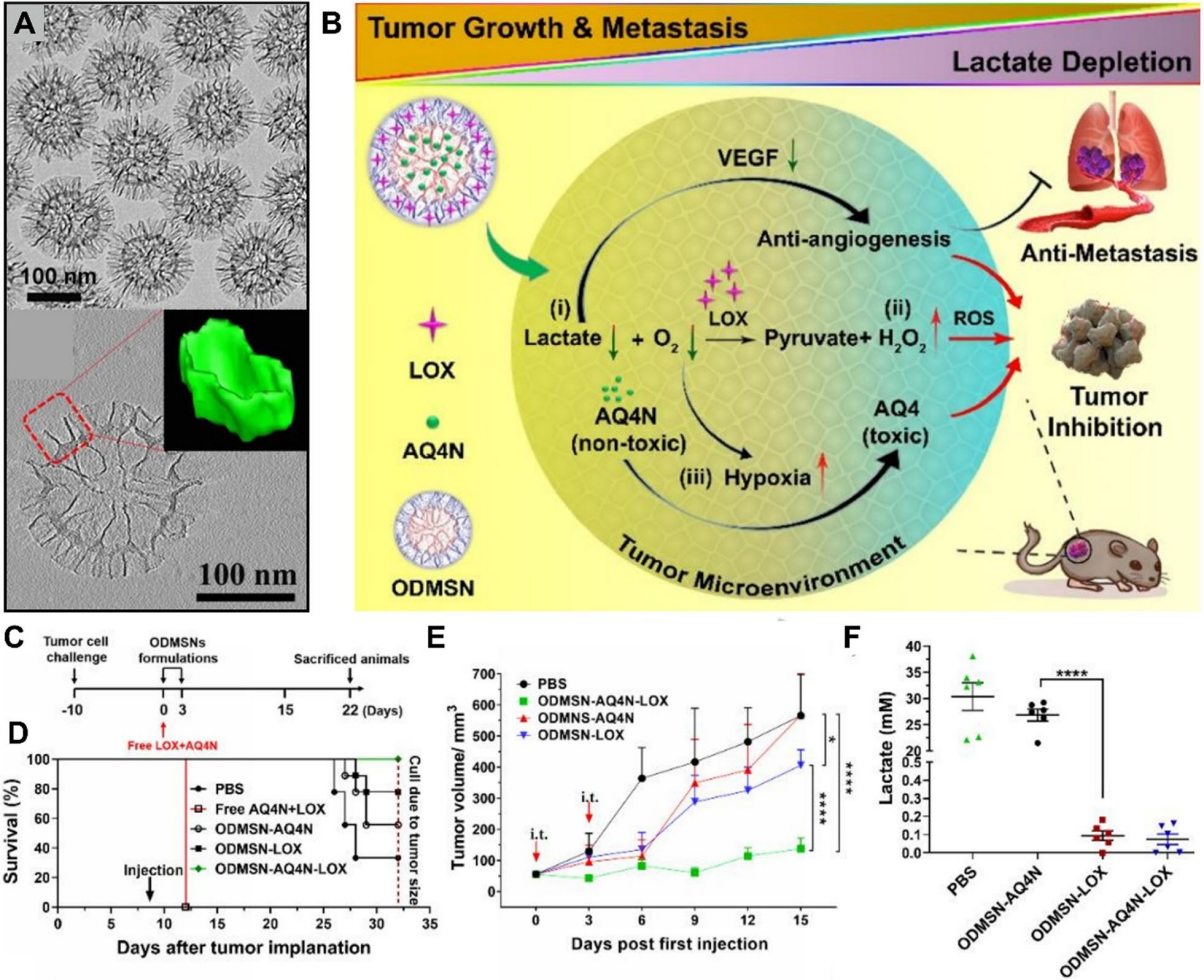
**A** TEM images of ODMSN. Inset shows the reconstructed 3D subunit. **B** Scheme of ODMSN nanoplatform induced combinational anti-cancer strategy. **C** The in vivo experimental design. **D** Survival curves of mice after treatments. **E** Tumor growth profile after treatments. **F** Intratumoral lactate concentration at 48 h after treatments. Reproduced with permission [123]. Copyright © 2020 Wiley‐VCH GmbH

A number of composite nanomaterials with inorganic nanoparticles as the core backbone have been explored in lactate attenuation and combinational anti-tumor therapy [123–127]. The advantages of inorganic mesoporous silica nanoparticles in tumor lactate attenuation and metastasis inhibition have been well proved by our group [123]. In our work, a unique mesoporous silica nanoparticle (named as ODMSN) was prepared. It featured an openworked core and a dendritic shell, both of which consist of silica nanosheets with thickness of $\sim$ 3 nm (Fig. 8). This unique architecture provided internal and external compartments that enabled high loading capacity and sequential delivery of large molecular LOX and a small molecular prodrug AQ4N (or banoxantrone). The LOX was adsorbed in the external dendritic compartment while the AQ4N molecules diffused into the inner core after loading process. The outer LOX served as gate for the inner AQ4N. Thus, this design achieved sequential release of LOX and AQ4N in cells. Moreover, due to the special nanostructure, the high loading capacity of LOX ($>0.7 \mathrm{g}/\mathrm{g}$) achieved excellent intratumoral lactate consumption performance ($>$99.9%), resulting in anti-metastasis and strengthened tumor hypoxia. The elevated hypoxia activated pro-drug AQ4N to its active form (AQ4) for potent chemotherapy.

Gao et al. demonstrated hollow MnO_2_ (HMnO_2_) nanoparticles (denoted PMLR) embedded with LOX and a glycolysis inhibitor 3PO (i.e., 3-(3-pyridinyl)-1-(4-pyridinyl)-2-propen-1-one) as another typical example for lactic acid exhaustion and immunotherapy [124]. The PMLR nanoparticles were coated with red blood cell membrane which benefited long-circulation and tumor targeting via CD47, a transmembrane protein that prevented internalization of macrophages. The lactic acid in TME was consumed by oxidation reaction catalysed via LOX. At the meantime, intracellularly released 3PO inhibited the production of lactate and ATP supply. The MnO_2_ nanoparticles decomposed H_2_O_2_ to O_2_ which sensitized both intracellular and extracellular processes. Consequently, the nanosystem effectively improved the antitumor effect in combination with immune checkpoint blockade therapy.

### Modulation of other signaling in glycolysis

As forementioned, hypoxia plays key roles in regulating tumor glycolysis. It has been reported that in situ oxygen generation via nanoparticles could relieve hypoxia and downregulate HIF-1 and c-Myc of both tumor cells and regulatory T cells, slowing down tumor progression [128, 129]. To date, many hypoxia targeting nanomedicines have been developed to fight against tumors, which have been well reviewed elsewhere [129–131]. Herein, we focus on other glycolytic signaling that have become have become targets for nanomodulation in tumor glycolysis, such as PDH, HK II, c-Myc and PI3K–AKT–mTORC1.

**Fig. 9:**
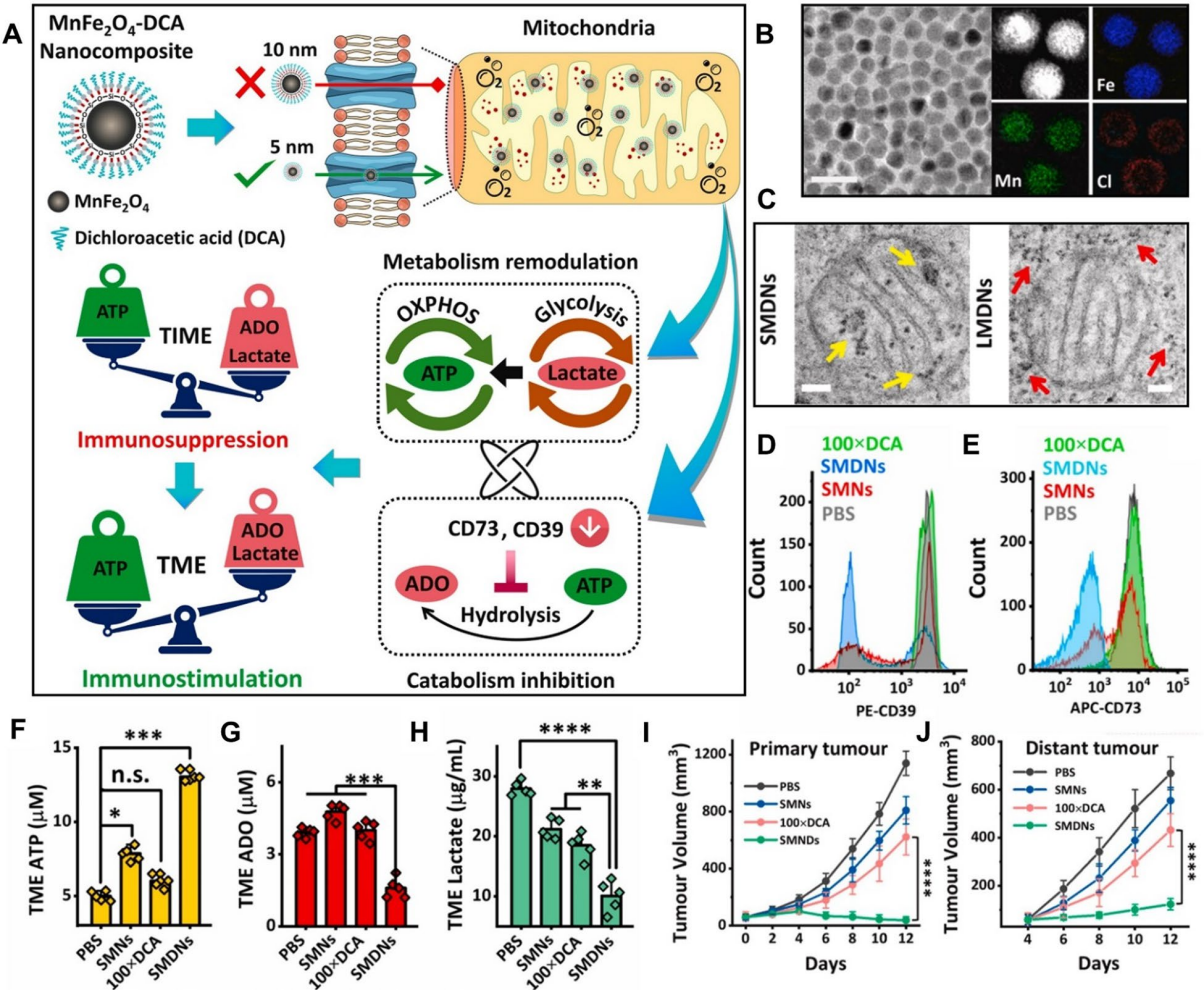
**A** Illustration of the mechanism of MnFe_2_O_4_-DCA nanocomposites (SMDNs) in modulation of immunosuppressive TME. **B** TEM image and elemental mapping of the nanocomposites. **C** Cryogenic TEM images of mitochondrion localized in 4T1 cells treated with small SMDNs (~ 6 nm) or counterpart large MnFe_2_O_4_-DCA nanocomposites (LMDNs, ~ 10 nm). Arrows point to nanoparticles. **D**, **E** The expression of CD39 and CD73 on 4T1 cells. **F–H** The level of ATP, ADO and lactate in TME. **I**, **J** Tumor growth profile in bilateral tumor model. Reproduced with permission [132]. Copyright © 2022 Elsevier Ltd

Our group reported an ultrasmall bimetallic oxide nanoparticle (MnFe_2_O_4_) functionalized with dichloroacetic acid (DCA) that is able to regulate tumor glycolysis and immunosuppressive TME via activating PDH located in mitochondria (Fig. 9A) [132]. DCA bore two missions in this nano-design. Firstly, it could activate PDH to enable a shift of tumor metabolism from glycolysis to OXPHOS, meanwhile suppressing lactate generation to relieve immunosuppression. Secondly, DCA inhibited the expression of HIF-1 and downregulated the downstream CD39 and CD73, leading to reduced catabolism of ATP and ADO, which further reverse the immunosuppressive TME. However, DCA not only exhibited poor bioavailability but also showed dose-dependent toxicity. The ultrasmall MnFe_2_O_4_ nanoparticles helped DCA to maximize its effects and overcome its limitations. The ultrasmall size enabled efficient DCA delivery into mitochondria via transition pores (size $\approx$ 6 nm) (Fig. 9B, C). At the meantime, MnFe_2_O_4_ decomposed intracellular H_2_O_2_ to release oxygen, thereby relieving hypoxia and improving the bioactivity of DCA. With the assistance of MnFe_2_O_4_, the nanocomposite dramatically inhibited the expression of immunosuppressive molecules including CD39, CD73, ADO and lactate while significantly increased ATP production (Fig. 9D–H). The efficacy was 100 times higher than that of free DCA. By reversing the immunosuppressive TME, this strategy effectively inhibited growth of primary and distal tumors as well as tumor metastasis. These findings are consistent with the results in another work, in which polymer nanoparticles containing a prodrug of DCA (Mito-DCA) significantly improved intratumoral lymphocytes infiltration and immunological activation [133].

**Fig. 10:**
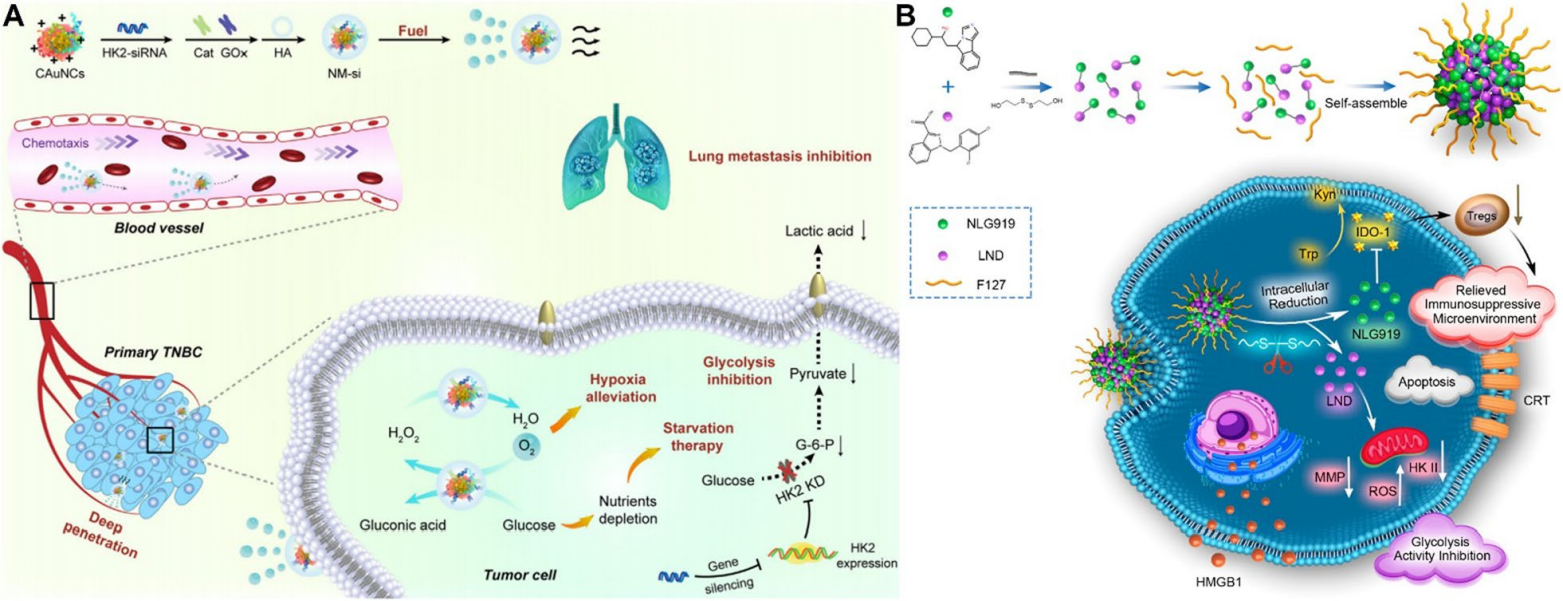
**A** The schematic preparation and functions of self-propelled gold-based nanomotor. Insert is TEM image of nanomotor. Reproduced with permission [134]. Copyright © 2021 Chinese Pharmaceutical Association and Institute of Materia Medica, Chinese Academy of Medical Sciences. Production and hosting by Elsevier B.V. **B** The scheme of the preparation and function mechanism of the GSH-responsive nano-prodrug co-delivering NLG919 and LND for regulation of tumor glycolysis and immunosuppression. Reproduced with permission [135]. Copyright © 2021, American Chemical Society

Yu et al. integrated HK II siRNA, GOx and catalase (CAT) on gold nanoclusters to construct a self-propelled nanomotor that reversed hypoxia and glycolysis for strengthened anti-metastasis (Fig. 10A) [134]. The GOx and CAT constructed a cascade enzymatic reaction. The H_2_O_2_ produced by the GOx catalysed oxidation of glucose was continuously converted to oxygen by CAT to alleviate hypoxia conditions. Interestingly, the persistently generated oxygen bubbles endowed nanomotor with faster movement and deeper tumor infiltration. Meanwhile, knockdown of HK II via siRNA significantly inhibited aerobic glycolysis which synergized with hypoxia alleviation to inhibit migration and invasion of 4T1 cells. It was further demonstrated that the in vivo lung metastasis of TNBC was significantly reduced with pre-treatment of nanomotors.

Recent studies indicated that HK II targeting strategies functioned synergistically with other signaling regulation in the alleviation of tumor immunosuppressive microenvironment. For example, Liu et al. demonstrated the lessened immunosuppression in a 4T1 tumor model by using a nanoprodrug consisting of inhibitors of HK II and indoleamine 2,3-dioxygenase (IDO-1) (Fig. 10B) [135]. The nanoprodrug was prepared by a F127-coated dimer that connected lonidamine (LND) and NLG919 by a disulfide bond. In response to excessive GSH in tumor, two inhibitors were released by the cleavage of disulfide bond. As a HK II inhibitor, the LND significant decreased the expression level of HK II to disrupt the phosphorylation of glucose to glucose-6P and limit the rate of glycolysis. Furthermore, LND mediated ROS generation via altering mitochondrial ultrastructure, effectively induced immunogenic cell death (ICD) upon the exhaustion of GSH by disulfide bond. The enzyme IDO-1 in tumor catalyzed the cleavage of L-tryptophan and production of kynurenine which promoted the function of immunosuppressive Tregs [136]. In Liu’s work, NLG919, a highly IDO-1-selective inhibitor [137, 138], has been applied to inhibit Tregs and restore the function of cytotoxic T lymphocytes. The tumor growth was dramatically suppressed by using this two-pronged nanotherapeutic strategy [135].

Simultaneous reduction of HK II and PD-L1 expression has been reported as another effective therapeutic strategy enabling dual regulation of tumor glycolysis and immune tolerance [139]. It is achieved in a supermolecular prodrug nanoplatform that can codeliver bromodomain-containing protein 4 inhibitor (BRD4i) JQ1 and pyropheophorbide a (PPa). JQ1 remarkably hindered the transcription of *c-Myc* and destabilize c-Myc protein to suppress glycolysis [140, 141], in which process the expression of HK II was reduced [139]. JQ1 bore another important function which downregulated PD-L1 [139, 142]. The role of PPa in the nanoplatform was to produce ROS and to promote activation of CD8+ T lymphocytes upon near-infrared laser irradiation. Collectively, these prodrug nanoparticles achieved excellent anti-tumor performance via inhibiting glycolysis, relieving immunosuppression and provoking anti-tumor T cell immunity.

PI3K–AKT–mTORC1 is also a good target for nanomedicine to regulate glycolysis. A recent report has demonstrated the interesting function of Realgar (a traditional Chinese medicine) nanoparticles on glucose metabolism reprogramming in cancer cells [143]. It has been shown that Nano-realgar could downregulate the expression of both HIF-1 and PI3K–AKT–mTORC1 in vitro and in vivo, showing the potential of Realgar in glycolysis-centred cancer therapy.

### Glycolytic reprogramming of tumor-associated immune cells

**Fig. 11:**
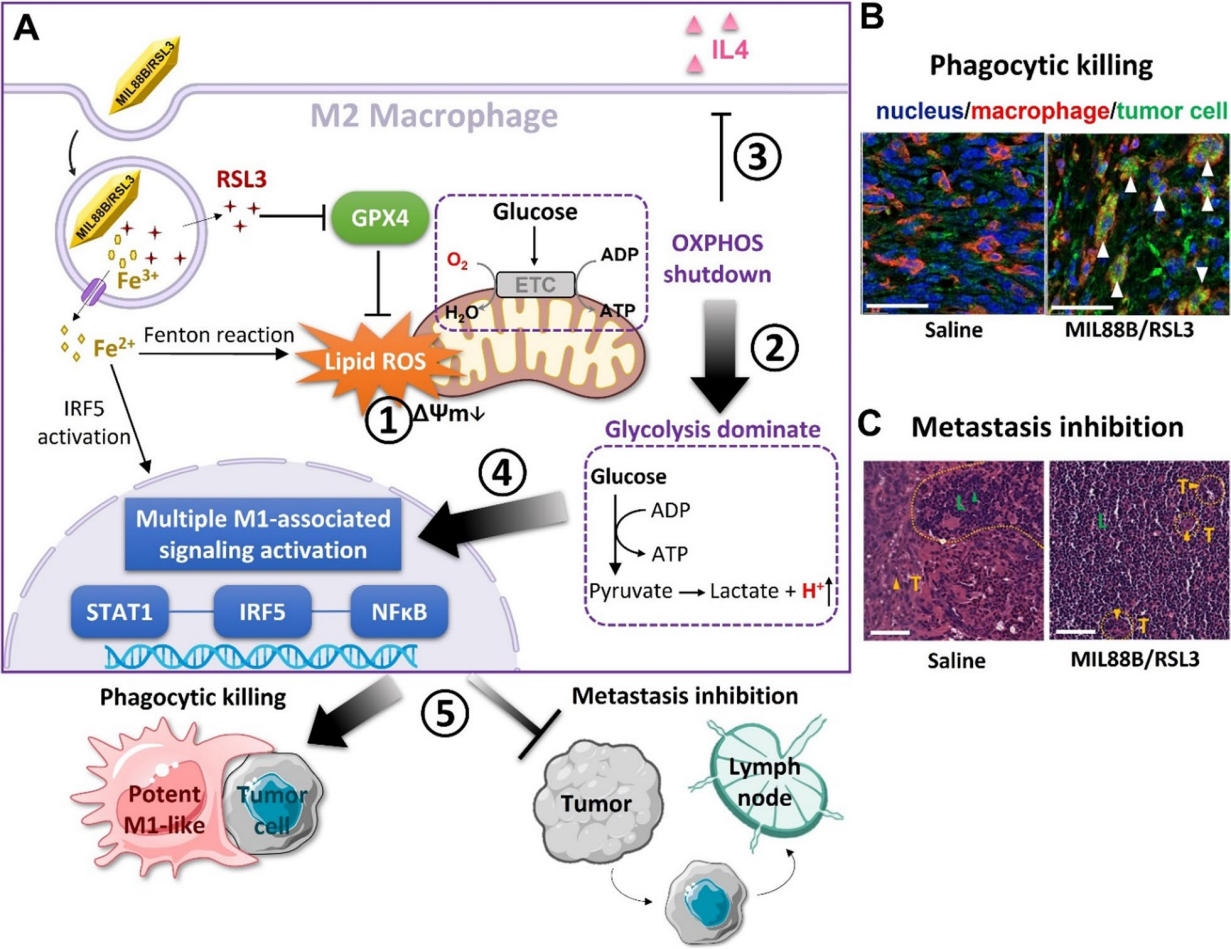
**A** Illustrated mechanism of ferroptosis-strengthened glycolytic programming and macrophage polarization via iron-based MOF nanoparticles (MIL88B/RSL3). **B** Confocal images of macrophage (red) and tumor cell (green) in tumor tissues. Arrows point to MIL88B/RSL3-induced phagocytosis of tumor cells by macrophages. **C** H&E staining of draining lymph node sections, showing the inhibition effect of MIL88B/RSL3 on metastasis. Yellow dashed lines distinguished the area of infiltrating tumor cells (T) from lymphocytes (L). Reproduced with permission [144]. Copyright © 2021 American Chemical Society

Due to the complicated metabolic interactions between tumor cells and immune cells within TME, there is emerging interest to regulate glycolytic metabolism of tumor-associated immune cells via nanotechnology for anti-tumor purposes. Very recently, our group reported a nano-design that achieved powerful anti-tumor polarization of macrophages via ferroptosis-strengthened metabolic and inflammatory regulation (Fig. 11A) [144]. Metabolic features play a key role in supporting macrophage phenotypes and functions [89, 145]. The metabolic profile of anti-tumor pro-inflammatory macrophages are distinct from pro-tumor anti-inflammatory macrophages. The former and latter relies on glycolysis and OXPHOS for energy demand, respectively. In this work, we have shown the nano-formulation consisting of iron-based MOF (MIL88B) and a ferroptosis activator (RSL3) could mediate lipid peroxidation to disrupt mitochondrial function. Consequently, the metabolic state of macrophages was shifted from OXPHOS to glycolysis, and the phenotypes are programmed from anti-inflammatory to pro-inflammatory. Furthermore, the intrinsic properties of iron species helped to drive inflammatory modulation, enabling reprogrammed macrophages possessing a high level of pro-inflammatory output that benefited anti-tumor activities. This regulation strategy not only elicited macrophage-mediated phagocytic kill effects on tumor cells but also dramatically inhibited tumor metastasis (Fig. 11B, C). Previous report has shown that macrophages competed with tumor epithelial cells for glucose [146]. This behavior significantly impacted on tumor metastasis. Our findings are consistent with this study, further supporting that targeting glycolytic metabolism in immune cells holds promise for anti-tumor purposes.

## Conclusion and future perspectives

Generally, we have summarized the origins and development of tumor glycolysis, then revealed the recent advances in glycolysis-associated regulatory strategies based on diverse nanotherapeutic platforms. The research progress shows that the unique advantages of nanoparticles empower biomolecular glycolytic modulators in antitumor applications. Nanoparticles can achieve well-controlled cargo delivery and release by fine-tuning the chemical composition and spatial structure. They are able to initiate cascade biochemical reactions with active biomolecules intracellularly or extracellularly to amplify the glycolytic metabolic modulation from multiple perspectives. More importantly, nanoparticles provide an appropriate platform for the integration of combination therapies such as chemotherapy, radiotherapy and immunotherapy, offering more opportunities to advance novel nanomedical applications. Despite the impressive achievements of nanomodulation in glycolysis, there are still many challenges and opportunities to be seized and addressed.

- *Exploiting the heterogeneity of cancer metabolism *Current studies have taken into account the differences in metabolite concentrations inside and outside tumor cells when designing nanomodulation modalities. However, it has been overlooked that the spatial distribution of some metabolites in solid tumors is often in a gradient manner and varies with the type of cancer, affecting the tissue distribution [147]. For example, the distribution of lactate and relevant transporters changes with the degree of hypoxia. Tumor cells in the core are usually more hypoxic and glycolytic due to the limited access to nutrients and oxygen [148]. Enhanced hypoxia and glycolytic conditions upregulate the expression of MCTS and GLUTs which in turn changes the metabolic environment [149–151]. As a result, metabolic features are heterogeneous rather than uniform across all cancers. It has been reported that the location of high GLUT1 and MCT4 expression in relation to distance from blood vessels is different in squamous cell carcinoma and adenocarcinoma [152]. Therefore, when designing nano formulations for different cancers, the penetration depth in tumor of the nano drug should match to the distribution of the target metabolites or receptors in order to maximize the therapeutic effect. In turn, by exploiting the heterogenous distribution of metabolites and receptors, nanotherapeutic agents are expected to target specific cell populations with enhanced precision. It is worth noting that there is growing evidences of heterogeneity in glycolytic metabolism across different tumors [153]. For example, dramatic elevated gene expression of OXPHOS was observed in patients of diffuse large B-cell lymphoma [154]. It implies that therapies targeting glycolysis may be only effective in tumors that are highly dependent on glycolysis. It is therefore necessary to take the heterogeneous nature of tumor metabolism into account when developing novel therapeutic approaches.
- *Exploring the dynamic metabolism upon nanomodulation *The view that cancer metabolism is flexible and context-specific is becoming widely accepted [155]. Tumor glycolytic metabolism is no exception, evolving during cancer progression [156, 157]. Tumor regulates metabolic pathways in response to their nutritional, biosynthetic and energy requirements, including altering dependence on OXPHOS [158]. The hallmarks of mitochondrial respiration have been found to be upregulated in cancer cells with more invasive and distal metastasis phenotype [159, 160]. However, the current understandings of nanoregulated glycolysis are mostly limited by oversimplified in vitro and in vivo experiments. The research into nanomedicines targeting tumor metabolism is still in infancy. Detailed characterization of dynamic metabolic changes in selective biological models before and after treatment is lacking but necessary, which would be helpful to judge whether the design and timing of nano-treatment is appropriate. Exploring the utilities of nanotherapeutics at different stage of tumor progression would be more informative to advance the future development of tumor metabolic therapies.
- *Regulating tumor glycolytic metabolism *via* immune cells* Accumulating evidence has demonstrated metabolic reprogramming of immune cells in tumors, which interact with tumor metabolic evolution [7, 77, 157]. In some cases, activated immune cells especially those with anti-tumor functions have metabolic demands similar to proliferating tumor cells. As mentioned earlier, both pro-inflammatory macrophages and cytotoxic CD8 + T cells require high level of glycolysis to maintain their phenotypes and functions, while their pro-tumor counterparts (i.e., anti-inflammatory macrophages and Tregs) are more dependent on OXPHOS.^6, 87^ Thus, glycolytic reprogramming of tumor-promoting immune cells may not only limit tumor growth through nutritional competition, but also create the conditions for reversing the immunosuppressive microenvironment. This scenario can be realized through the well-designed nanomedicines with precise targeting and biochemical regulation properties. In-depth investigation of the interplay between metabolism and immunity at the nano-bio interface is expected to drive the development of advanced anti-tumor therapeutic strategies. Hence, the development of novel nanoplatforms with intrinsic dual regulatory functions in both metabolism and immunity is worth of further investigation with more efforts.
- *Investigating combined therapeutic strategies* As glycolysis involves multiple biosynthetic pathways within tumor cells, interrupting glycolysis may make the tumor more vulnerable and sensitive to other available treatments. For example, tumor glycolysis involves in the induction of drug resistance through multiple mechanisms, such as induction of EMT and autophagy [161]. It has been shown that a large number of glycolytic enzymes or intermediates contribute to drug resistance [161]. Therefore, it is speculated that therapies targeting glycolysis are appropriate in combination with chemotherapy. In situ oxygen or radical production via photothermal/photodynamic/chemodynamic therapies has high chance to synergize with glycolysis targeting nanomedicine [162–164]. It is therefore worth exploring more advanced combinational therapies to improve anti-tumor efficacy.

## Declarations

### Data availability

Data sharing not applicable to this article as no datasets were generated or analysed during the current study.
